# Dietetical and Medical Hydrology

**Published:** 1850-05

**Authors:** 


*Dietetical and Medical Hydrology. A Treatise on Baths; including Cold, Sea, Warm, Hot, Vapor, Gas, and Mud Baths; also, on the Watery Regimen, Hydropathy, and Pulmonary Inhalation: with a description of Bathing in Ancient and Modern Times.* By John Bell, M. D., Member of the American Medical Association, &c. &c. Philadelphia: Barrington & Haswell. 1850. 12mo. pp. 658, including a very copious index.

The name of the author of this admirable volume has been too long and too honorably associated with the subject of which it treats, to need any special introduction to notice at our hands. It would be natural, without such a previous reputation, to look for something that would do honor to the American Medical Press, from an editor, lecturer and writer of his veteran experience and standing; nor will the reader be disappointed in his expectations.

In the particular department of "Hydrology," Dr. Bell has long been without a rival. In the midst of crowds of self-sufficient hydropathists, and a few worthier but less importunate competitors, his original essay has maintained its place above them all, as the only classical authority. The hygienic and therapeutic precepts of his "Baths and Mineral Waters" had become established laws amongst us, many years before the blundering charlatanisms of Preissnitz, and his imitators, had emerged from their appropriate obscurity. In short, no one who knows any thing of the leading American medical books, can fail to recognize, in the title page above, many topics that must pleasantly remind him of a well-tried and valued monitor, under whose guidance even the unprofessional and uninitiated reader would have little to learn, and less to fear, from the new-fangled empiricisms of the Græfenberg pretenders.

The new treatise, however, is neither in scope, objects or extent, a mere reprint of the old. A very cursory examination of its pages will demonstrate, what the author tells us in his preface, that he has "enlarged on all the topics of his former work," and "has added new ones—so far as relates to baths and bathing," while mineral waters are left out to "form the subject of a separate volume." His endeavor was "to exhibit a systematic view of the operation and effects of the different kinds of baths on the animal economy, as well in its healthy as its diseased states."

The book is intended for all classes of readers, although the general student is studiously warned that he will find much that he can not and ought not, as an unprofessional man, to be interested in; while there is abundance left that must be regarded as property common to all. We cannot help viewing such a combination as too strongly savoring of the hybrid in its character and influence, to increase the scientific value of the work. The attempt to serve two very different parties, in this manner, under the same cover, with dishes that may be unpalatable to the one and unwholesome to the other, must be at all times an experiment, to say the least of it, of doubtful policy. We would be glad, therefore, for many reasons which need not be dwelt upon at length, to see the popular portions of the book entirely separated from the strictly medical, and given to the public unencumbered with what can prove harmless as well as interesting and useful to the professional reader only. Still, for the sake of the great benefit the intelligible precepts and peculiar information will confer upon all who may be induced to read them, we are extremely thankful to our author for his book precisely as it is. We would hail it gladly in its present shape, and cheer it onwards in its course, were it only for the desire its author manifests to contribute towards the "increase of the public health and of individual comfort and pleasure." These philanthropic interests, he thinks, would be powerfully promoted by bringing into greater vogue the practice of bathing, to the study of which, in all its aspects, his pages are devoted. He has our most cordial wishes in behalf of so laudable an ambition; and we shall be most happy if any sympathy of ours can aid him in pressing on a consummation which is notoriously and most lamentably needed in the land.

It would be impossible, within the space to which the present sketch is necessarily confined, to do justice to the merits of the work in an extended appreciation of its principles and facts, or to attempt to discuss the particular tenets of the author. We must restrict ourselves, therefore, to a brief enumeration of the leading topics of the different chapters, as they appear in regular order of succession.

After some preliminary remarks in regard to the neglect of bathing in England and the United States, and upon the structure and functions of the skin, together with the proper care of this latter in its relations to clothing, exercise and cleanliness, he enters *in extenso* on the history of bathing in ancient and modern times, and among different people. Then comes the individual consideration of Baths, in their varieties, nature, materials and hygienic management. These topics occupy the first fourteen chapters. With Chapter 15th begins the study of the "Watery Regimen," which is continued in a very instructive and entertaining manner throughout the nine succeeding chapters. In Chapters 25th, 26th, 27th and 28th, we are favored with a full account of Hydropathy, which will be read with great interest and advantage by the skeptical as well as the believers in that would-be system of the healing art.

The five chapters next in order are devoted to the Cold Bath in each of its numerous and important relations; among which its therapeutic uses in febrile, exanthematous, hemorrhagic, neuralgic and other diseases of different types and tissues, are fully shown. Then, in the course of Chapters 30th, 34th, 35th, 36th and 37th, we have Sea Bathing under all its aspects. Upon this subject, it may be as well to remark, that Dr. Bell has freely availed himself of the tract of M. Gaudet, who as medical inspector of the baths at Dieppe for a period of eleven years, had acquired a great deal of valuable experience, the results of which are not to be found elsewhere.

The Warm Bath comes next in order of succession, and is presented to us, under a great variety of heads, in five very interesting chapters. The Hot Bath succeeds the warm bath in two equally instructive chapters, and is followed in five more, including the 50th, by the account of the Vapour, Hot Air and Fumigation Baths, and their varieties, uses and effects. In chapters 51st, 52d and 53d, we have the subject of "Pulmonary Atmiatry," or Inhalation in all its forms, including that of ether and chloroform. The three concluding chapters are occupied with the consideration of the different kinds of Medicated Baths; under which are included natural and artificial mineral waters, mud baths, gas baths, iodine, mineral acid, compressed air and common air baths. A copious and unusually complete index very appropriately concludes the volume. We take leave of the whole work with regret; not merely on account of the pleasure experienced in its perusal, but because its intrinsic excellence inclines us to pay it more respect than can be shown in the present meagre notice. If the man who makes two blades of grass grow where only one grew before, be a benefactor to his race, surely he deserves well of his countrymen who bestows upon them a genuine new book. How much heavier the obligation, then, if that book brings agreeably and forcibly before the general mind a matter which comes home to the physical and moral interests of every member of society.

These are our sentiments, and they lead us once more to thank Dr. Bell for his unique production. We receive it not only as a boon and an honor to the American Profession, and to our countrymen in general, but as a tribute to humanity at large. And we are happy to know that it comes as the forerunner of another gift from one whose public and private life have long since stamped him as a true lover of his fellow-men, and an able as well as earnest advocate of all that is likely to improve their physical and mental being.

